# Multispectral Fluorescence Imaging for Fast Identification of Cold Stress in Pepper Plants

**DOI:** 10.3390/s26061799

**Published:** 2026-03-12

**Authors:** Reza Adhitama Putra Hernanda, Whanjo Jung, Me-Hea Park, Hoonsoo Lee

**Affiliations:** 1 Department of Biosystems Engineering, College of Agriculture, Life, and Environment Sciences, Chungbuk National University, Cheongju 28644, Republic of Korea; reza.a.p@chungbuk.ac.kr (R.A.P.H.); hwanzo758@chungbuk.ac.kr (W.J.); 2 Vegetable Research Division, National Institute of Horticultural and Herbal Science, Wanju 55365, Republic of Korea; poemmich@korea.kr

**Keywords:** *Capsicum annuum* L., deep learning, gated recurrent unit, nondestructive, plant phenotyping, snapshot multispectral imaging

## Abstract

This paper investigated the feasibility of snapshot multispectral fluorescence imaging for nondestructive identification of cold stress in pepper plants. Fluorescence spectra were obtained by exciting the plant with a 405 nm ultraviolet LED. The plants were grown under three temperature conditions: 17 °C (control), 10 °C (moderate cold stress), and 5 °C (severe cold stress). Raw fluorescence spectra extracted from the demosaiced snapshot images were used as inputs for a deep-learning pipeline consisting of feature extraction, an encoder–decoder GRU, and a multilayer perceptron (MLP), and the results were compared with conventional machine learning classifiers, including linear discriminant analysis (LDA), quadratic discriminant analysis (QDA), and a Gaussian support vector machine (G-SVM). Tukey’s HSD test indicated that the proposed deep-learning model achieved the highest cross-validation accuracy and consistently produced superior classification metrics (accuracy of 85.7%, precision of 85.3%, recall of 85.3%, F1-score of 85.2). The trained model was further applied to hyperspectral cubes to generate classification maps; however, moderate misclassification was observed, consistent with the overall prediction performance.

## 1. Introduction

Pepper, or chili (*Capsicum annuum* L.), belongs to the *Solanaceae* family and is an important agricultural crop with distinctive sensory attributes, particularly pungency, which is primarily attributed to capsaicin [1]. In spite of the pungent flavor, evidence have reported that pepper contains substantial levels of bioactive compounds, including capsaicinoids, carotenoids, flavonoids, tocopherols, and ascorbic acid, which confer benefits to human health [2]. Although pepper is native to South America, it has long been an integral part of Korean culinary culture, particularly as a key ingredient in *gochujang* (Korean red pepper paste) [3].

Korea’s temperate climate, characterized by four distinct seasons, poses particular challenges for pepper cultivation, especially during winter and the transition to spring [4]. Pepper plants are highly susceptible to low-temperature stress, which leads to cellular damage and reduced photosynthetic activity [5]. Accordingly, transcriptomic and metabolomic investigations have reported extensive molecular responses to cold exposure, identifying more than 10,000 differentially expressed genes and multiple hormone-related changes across two pepper cultivars [6,7]. Although transcriptome and metabolome analyses are highly sensitive and can elucidate specific biochemical alterations under stress, they require careful sample preparation and chemical reagents, limiting their practicality for large-scale monitoring. Therefore, there is an urgent need for alternative approaches that support conventional plant phenotyping while enabling rapid and scalable assessment.

Nondestructive evaluation (NDE) using imaging techniques has emerged as one of the most suitable approaches for plant phenotyping, encompassing both close-range imaging and remote-sensing applications. For instance, RGB cameras have recently been applied to detect freeze damage in strawberry [8] and lettuce [9]. However, RGB imaging is largely limited to external color and visual symptoms. In contrast, other NDE approaches, namely X-ray phase-contrast imaging [10], micro-X-ray fluorescence [11], thermal imaging [12,13], and hyperspectral imaging [14] provide more advanced assessments by capturing deeper relevant information. Among them, hyperspectral imaging has attracted substantial attention due to its versatility for plant sensing at both close- or wide-range (e.g., uncrewed aerial vehicles). In addition, hyperspectral imaging captures simultaneous spatial information across the continuous bands, typically spanning from 400 to 2500 nm. This rich spectral information enables the characterization of plant chemical constituents and the detection of stress-induced changes associated with varying environmental conditions.

Yet, the utilization of hyperspectral imaging remains limited by several practical limitations, including high instrument cost and relatively long acquisition times. To address these challenges while retaining the benefits of spectral imaging, multispectral cameras have emerged as a promising alternative. Multispectral cameras, on the other hand, capture a limited number of narrow spectral bands, enabling faster acquisition and a lightweight design (typically suitable for unmanned aerial vehicle platforms). Unlike line-scan hyperspectral imaging systems, where the incident light is dispersed by dispersive elements (e.g., prism–grating–prism or Offner imaging spectrographs) to obtain a continuous spectrum as a function of wavelength, multispectral cameras commonly use beam splitters, rotating filter wheels, or electronically tunable filters to acquire spectral information [15]. These optical approaches produce discrete spectral bands, usually spanning the visible (blue and green), red-edge, and near-infrared region. Such multispectral imaging systems have been widely explored for high-throughput plant phenotyping [16,17,18,19].

Even though these multispectral imaging approaches offer advantages over line-scan hyperspectral imaging, they still have limitations. When acquisition time remains a major concern, particularly due to the rotating filter wheel, and an expanded set of discrete wavelengths are still needed to preserve the chemical information of a scene (e.g., plant canopies), snapshot-based multispectral imaging can be a promising alternative. Snapshot multispectral imaging follows a concept analogous to the Bayer filter mosaic used in RGB cameras [20], enabling the acquisition of multiple spectral bands in a single exposure and therefore substantially reducing acquisition time [21,22]. In plant phenotyping applications, snapshot multispectral systems have demonstrated encouraging performance. For example, prediction models developed using snapshot multispectral data achieved an R-square of 0.7 with root mean squared error of 1.611 t/ha and 1.051 for aboveground biomass (AGB) and leaf area index (LAI) in rice [23], as well as in refs. [24,25,26].

While those studies used reflectance mode, fluorescence mode can also be applied in plant phenotyping. Fluorescence occurs when electrons absorb incident light, become excited to a higher energy state, and then rapidly return to the ground state while emitting light. In practice, this can be achieved by using an excitation light source (e.g., an ultraviolet (UV) lamp) at an appropriate wavelength, which induces the leaf to emit a fluorescence signal. Not all materials can emit fluorescence (i.e., contain fluorophores); however, plant leaves can do so because they contain chlorophyll, a natural fluorophore. Moreover, chlorophyll fluorescence is sensitive to physiological changes and has therefore been widely used for plant stress detection [27,28]. Accordingly, many studies have developed chlorophyll fluorescence imaging systems or chlorophyll fluorometers for stress assessment [27,29,30,31]. Nevertheless, many chlorophyll fluorescence approaches require dark adaptation prior to measurement; for example, these studies used a 20 min dark-adaptation period. Despite the high spatial resolution achievable with chlorophyll fluorescence imaging, this acquisition requirement can limit large-scale deployment (e.g., field applications). Therefore, fluorescence multispectral imaging using UV excitation could be a practical alternative for plant stress detection.

In recent years, numerous studies have focused on developing novel regression and classification methods based on deep-learning applied to spectroscopic data. Deep learning can be regarded as an advanced extension of chemometric techniques, as it enables automatic feature extraction through convolutional layers [32,33]. Although deep-learning was initially proposed to address image classification problems, its application in the realm of spectroscopy has demonstrated substantial potential, with steadily increasing adoption in recent years. For readers who are new to deep-learning-based spectroscopic modeling, a comprehensive tutorial is provided in ref. [32]. In the aforementioned study, a deep learning model was developed by [24], in which one-dimensional spectral data were first transformed into two-dimensional recurrence plots. The proposed approach achieved an overall accuracy ranging from 70% to 85% for heat stress detection in garlic plants. In our previous works, we developed a lightweight one-dimensional convolutional neural network (1D-CNN) model for food safety and quality assurance in novel food products, demonstrating excellent predictive performance [34,35]. Furthermore, an autoencoder-based deep learning model was proposed for spectral correction and model transfer, resulting in improved prediction accuracy for soluble solid content [36]. Likewise, a deep learning architecture known as 1D-SP-Net [37] achieved the highest accuracy (96.3%) when compared with partial least-squares discriminant analysis, random forest, and common 1D-CNN models.

Many conventional chemometric and deep-learning approaches implicitly treat spectral features as an unordered set of variables. In this paper, we propose a sequence-based deep-learning approach that explicitly models spectra as ordered sequential data along wavelength by implementing an encoder–decoder recurrent neural network (RNN) architecture, as proposed by ref. [38]. RNNs are well suited for learning dependencies in sequential signals, enabling the model to capture relationships among neighboring and distant wavelengths [39] and it helps to mitigate the vanishing-gradient problem [40]. Although encoder–decoder RNN frameworks have been explored in other domains [41], their application to spectral data in plant phenotyping, particularly for cold-stress detection in pepper plants using UV-induced fluorescence multispectral imaging, has not yet been reported. Based on these considerations, the main objective of this paper includes the following:

(i) Implement a snapshot multispectral imaging system with an ultraviolet illumination source to induce leaf fluorescence under three temperature treatments, and
(ii) Develop a one-dimensional deep learning-assisted classification model based on an encoder–decoder RNN. For benchmarking, classical machine learning methods, including linear discriminant analysis (LDA), quadratic discriminant analysis (QDA), and Gaussian support vector machine (G-SVM), were also evaluated.

The structure of this paper is organized as follows. Section 2 describes the establishment of cold-stressed pepper plants, data acquisition using a snapshot multispectral camera, the image-processing workflow up to spectral extraction, and the development of the proposed models. Section 3 presents and discusses the results. Finally, Section 4 concludes the paper with key findings and remarks.

## 2. Materials and Methods

### 2.1. Cold Stress in Pepper Plants

**Figure 1:**
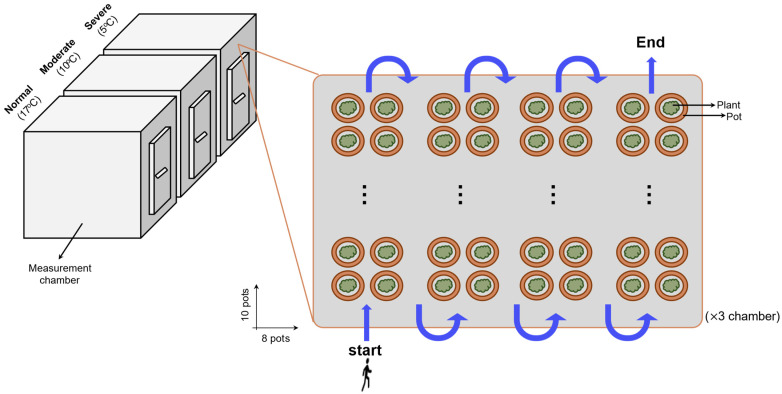
Experimental design for normal and cold-stressed pepper plants.

In this study, 240 Korean pepper plants (14 weeks old) grown in a commercial lightweight potting medium were used. Before the temperature treatments, all plants were acclimated for approximately two weeks under normal conditions (23/17 °C, day/night). The plants were then exposed to three temperature regimes (Figure 1): 23/17 °C (normal), 15/10 °C (moderate), and 10/5 °C (severe), in a measurement chamber at the Extreme Weather Research Center, Rural Development Administration (Jeonju, Jeonbuk Province, Republic of Korea) for a week experimental period under 70% relative humidity, a 12/12 h light/dark photoperiods, and regular irrigation. Each temperature treatment included 80 independent biological replicates.

### 2.2. Snapshot Multispectral Camera

**Figure 2:**
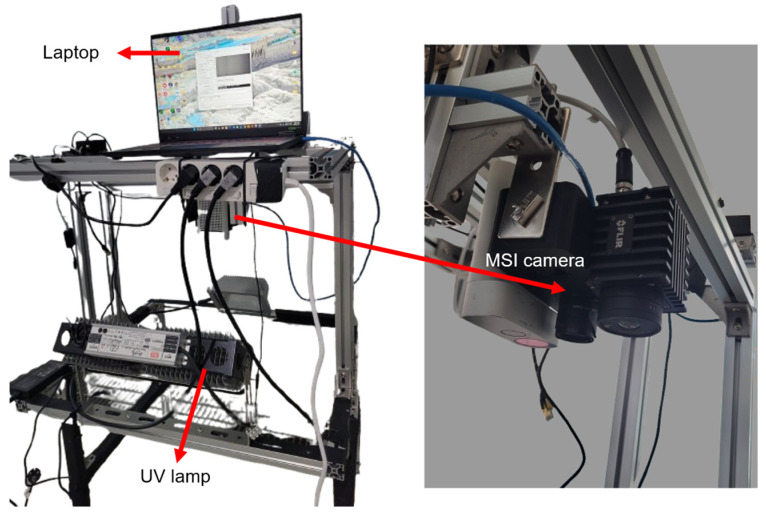
Computer vision system based on the snapshot multispectral fluorescence imaging for nondestructive identification of cold stress in pepper plants.

A basic computer vision system, comprising optical instrumentation, an illumination unit, and a personal computer, was used in this study (Figure 2). Multispectral images of pepper plants under control, moderate, and severe stress conditions were acquired using a snapshot-based multispectral camera (OCI-D2000; BaySpec Inc., San Jose, CA, USA) operating in the 603–870 nm spectral region. To capture leaf fluorescence intensity, the illumination system consisted of a 405 nm ultraviolet/blue light-emitting diode (LED) excitation source. This excitation wavelength was selected based on evidence that chlorophyll autofluorescence can be effectively elicited under 405 nm excitation [27]. Camera setting parameters, i.e., exposure time (1 s), and the data acquisition were managed through the Bayspec SpecGrabber 1100 software (Bayspec Inc., San Jose, CA, USA) operated in the ASUS TUF Gaming A16 (FA608) laptop (ASUSTeK Computer Inc., Seoul, Republic of Korea).

**Figure 3:**
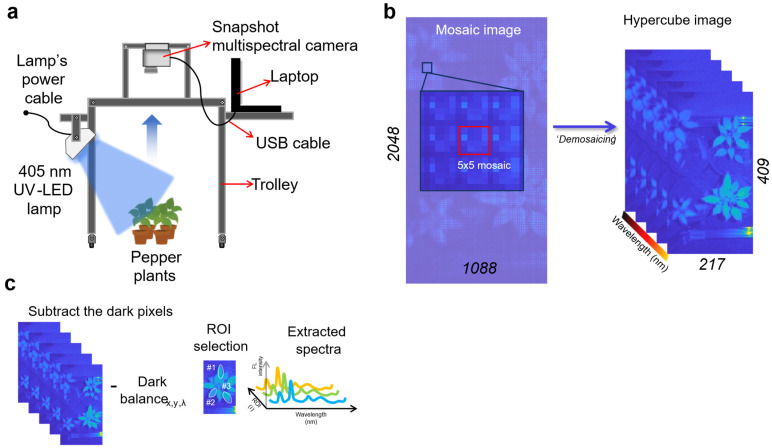
Multispectral imaging system mounted on a trolley for capturing up to four pots (**a**), snapshot spectral image demosaicing (**b**), and fluorescence hypercube correction and spectral extraction (**c**).

As the pepper plants were cultivated inside a growth chamber and the measurement protocol required non-moving samples, a manually operated trolley was fabricated in our laboratory to support and position the computer vision system (Figure 3a). The working distance between the plant canopy and the camera lens was maintained at approximately 50–60 cm, enabling the simultaneous measurement of up to four pots per snapshot. Experiments were conducted over six days, with image acquisition performed daily at 6 a.m. (local time) by turning off the chambers’ lamp and only keeping the UV LED turned on.

### 2.3. Snapshot Image Demosaicing, Hypercube Correction, and Spectral Extraction

In a single snapshot spectral image, pixels from multiple wavelengths are interleaved in a mosaic pattern; therefore, they must be reconstructed to obtain a complete hyperspectral (hypercube) image [42]. This reconstruction process is referred to as demosaicing (Figure 3b). The snapshot multispectral camera produced a mosaiced image of 2048 × 1088 pixels with 5 × 5 multispectral patterns. Based on the manufacturer’s specifications, using offsets of 0 and 3 along the x- and y-axes, respectively, a final 3D hypercube of 409 × 217 spatial pixels × 25 wavelengths were generated. After constructing the hypercube, fluorescence correction (Figure 3c) was performed by subtracting the dark reference from the raw hypercube, as described by the following equation.

(1) $${\mathrm{Ic}}_{\left(Z\right)}={\mathrm{Ir}}_{\left(Z\right)}-{\mathrm{Id}}_{\left(Z\right)},$$

In Equation (1), Ic, Ir, and Id correspond to the corrected, raw, and dark hypercube images. In addition, the correction was performed across the Z direction (bands).

Prior spectral modeling, the fluorescence spectra was extracted from the hypercube images utilizing the ‘polyroi’ function in MATLAB (R2023b; The Math Works Inc., Natick, MA, USA). For each pot, spectra were collected from three randomly selected leaves (from one pot), and the averaged spectrum was used as the representative sample. Spectra from normal plants were labeled as ‘0’, whereas those from moderately and severely stressed plants were labeled as ‘1’ and ‘2’, respectively, prior to model calibration. However, due to the data errors, e.g., saturated pixels and technical errors, this process resulted in a final dataset comprising 2385 spectra across 25 bands, which was compiled and stored in an Excel (Microsoft 365; Microsoft Corporation, Redmond, WA, USA) file for subsequent analysis.

### 2.4. Outlier Detection by PCA-SPE/DModX

**Figure 4:**
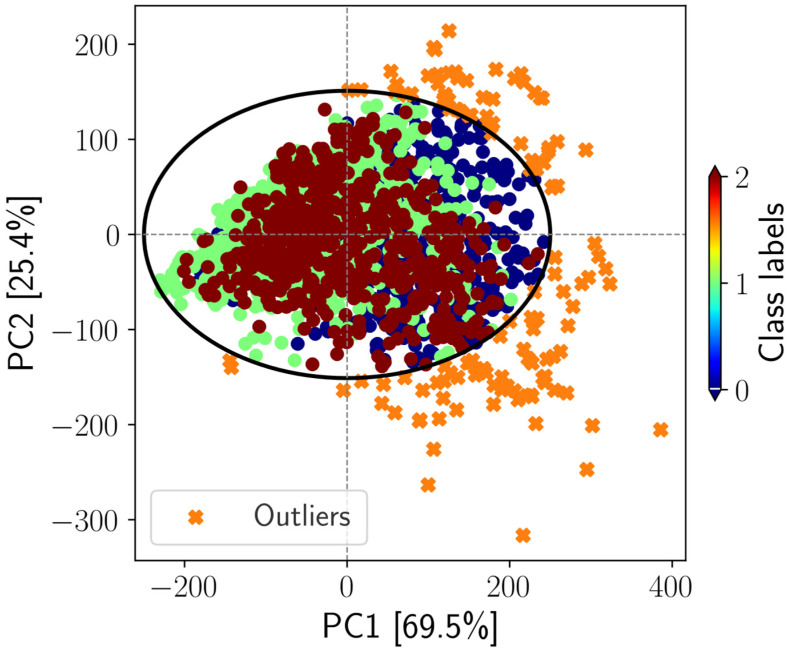
SPE/DmodX-based outlier detection projected onto the first two principal components (94.9% cumulative explained variance). The black ellipse represents the decision boundary used to classify observations as non-outliers or outliers. Original data refers to any spectral data at the *i*-th sample satisfies the decision boundary.

Outliers can adversely affect the predictive performance of the subsequent classification models [43]. Therefore, outlier detection was conducted using the following strategy. Principal component analysis (PCA) was implemented in Python using the open-source ‘pca’ package version 2.10.2 (https://github.com/erdogant/pca/, assessed on 15 January 2026) and applied to 2385 spectra measured across 25 bands; the first two principal components (PCs) explained 94.9% of the total variance (Figure 4). Beyond its role as an unsupervised exploratory tool in spectroscopic and chemometric domains [44,45,46], PCA is also effective for detecting atypical samples within multivariate datasets. The PCA score space was subsequently evaluated using squared prediction error/distance to the model (SPE/DModX) [47] with a threshold of ±2.5 standard deviations to identify potential outliers. As shown in Figure 4, samples located outside the decision boundary were classified as outliers; in total, 125 spectra (0 = 82 spectra, 1 = 21 spectra, and 2 = 22 spectra) were removed from the original dataset. Consequently, 2260 spectra across 25 bands were retained for the subsequent analyses.

### 2.5. Sample Selection According to the Similarity

Among the 2260 spectra measured across 25 bands spanning from 603 to 870 nm, sample selection based on similarity [48], i.e., SPXY algorithm [49], was used to split the dataset into calibration and prediction sets for model development. Given the *x_i_* = (*x_i1_*, *x_i2_*, …, *x_iZ_*) and *x_j_* = (*x_j1_*, *x_j2_*, …, *x_jZ_*) denote the spectral vectors of the *i*-th and *j*-th sample subsets, respectively, and let *y* be the reference vector containing the predefined numerical labels. Because SPXY considers the Euclidean distances (*D*) in both the *x* and *y*, the distances can be expressed as shown in Equations (2) and (3).

(2) $$D_{x}(i,j)=\sqrt{\sum_{k=1}^{Z}{\left(x_{i,k}-x_{j,k}\right)}^{2}},$$

(3) $$D_{y}(i,j)=\left|y_{i}-y_{j}\right|,$$

**Figure 5:**
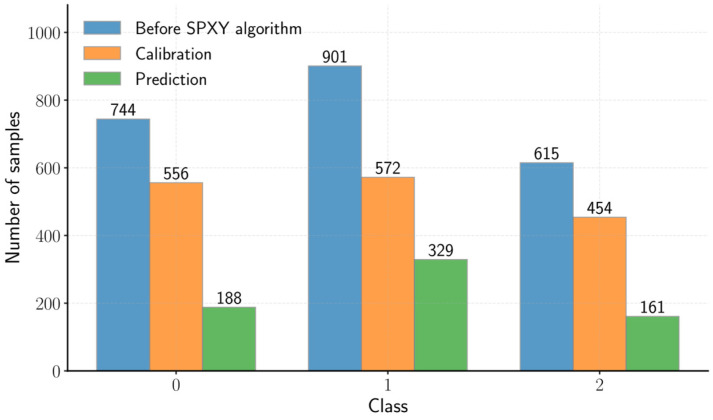
Class distribution before and after SPXY algorithm.

Thus, by combining both distance measures, the SPXY subset distance *D_SPXY_* is computed as defined in Equation (4). Finally, in the present study, the dataset was partitioned into calibration and prediction sets at a 7:3 ratio. The distribution of classes in both the calibration and prediction sets is illustrated in Figure 5.

(4) $$D_{\mathrm{SPXY}}(i,j)=\frac{D_{x}(i,j)}{\max D_{x}}+\frac{D_{y}(i,j)}{\max D_{y}}$$

According to Figure 5, some classes did not strictly follow the intended splitting ratio. For example, class ‘0’ contained 744 spectra in total, and after splitting, nearly 75% of these were assigned to the calibration dataset. This discrepancy was likely caused by the application of SPXY as a global splitting method, rather than a stratified splitting approach within each class.

### 2.6. Classification Model Development

During the preliminary study, various spectral preprocessing techniques, as reported in a review study [10] (e.g., SNV, MSC, and Savitzky–Golay derivatives), were evaluated; however, none of these approaches improved the model accuracy. Therefore, only the raw fluorescence spectra were used for model development. Subsequently, classical machine-learning methods were employed to develop discrimination models based on the fluorescence spectral data, including linear discriminant analysis (LDA), quadratic discriminant analysis (QDA), and Gaussian support vector machine (G-SVM). The LDA and QDA models were developed using the default settings provided by the ‘Scikit-Learn’ library [50], whereas the G-SVM model was optimized using a genetic algorithm with 5-fold cross-validation, yielding optimal hyperparameters of 6.98 (gamma) and 17.66 (regularization).

**Figure 6:**
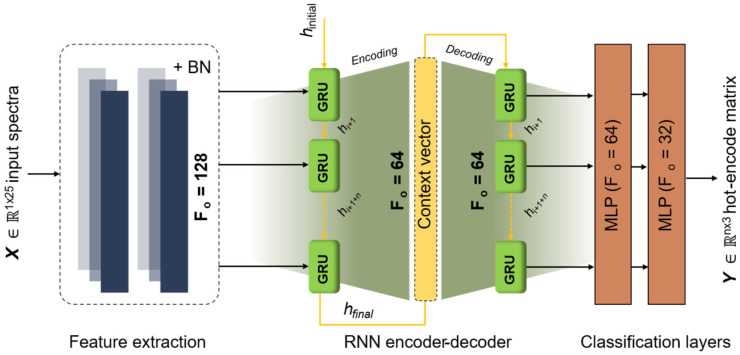
Deep-learning architecture for cold-stress detection based on fluorescence spectra based on ref. [41]. In this schematic diagram, BN refers to batch normalization, F_i_ and F_o_ are the number of input and outputs from a certain layer, GRU denotes the gate recurrent unit, *h* is the hidden state, and MLP is multilayer perceptron. The mathematical concept of GRU can refer to [51].

Besides applying three machine-learning techniques, we also propose a deep-learning model, as illustrated in Figure 6. Compared with conventional methods, deep-learning enables automatic feature extraction. As shown in Figure 6, the proposed architecture follows the framework in ref. [41] and comprises three main components: (i) deep feature extraction, (ii) a recurrent neural network (RNN) encoding-decoding layer, and (iii) multilayer perceptron (MLP). Given an input spectral feature vector consisting of 25 bands, feature extraction is performed using a one-dimensional convolutional neural network (1D-CNN). In this stage, double convolutional layers equipped with batch normalization (BN) are applied to stabilize training by standardizing the feature distribution [41]. The convolutional layer uses a kernel size of 2, stride of 1, ‘same’ padding, and ‘He-normal’ initialization. Furthermore, the encoder–decoder RNN generates a fixed-length context vector (encoder) and then uses this vector to produce a sequential representation of the input variables (decoder). In this case, we used a gate recurrent unit (GRU) as encoder and decoder [38]. Finally, two MLPs with 64 and 32 neurons are used, followed by a Softmax classifier for multi-class prediction. In addition, a rectified linear unit a.k.a. ReLU activation is applied throughout the network prior to the Softmax output layer. The detailed deep learning configuration is listed in Table 1.

Before training the deep-learning models, the reference label vector containing numeric class was converted into a one-hot encoded matrix to match the multiclass classification setting. Furthermore, the calibration dataset, comprising spectral inputs and their corresponding one-hot labels, was split into training and validation subsets at a 4:1 ratio using stratified sampling, with ‘random_state’ fixed to 42 to ensure reproducibility if repeated runs are required. Because the proposed approach addresses a multiclass classification problem, categorical cross-entropy was used as the loss function. To mitigate overfitting due to the excessive learning of the planned maximum of 500 epochs, we applied the following regularization strategies: (i) early stopping monitored on validation loss, with a minimum delta of 10^−4^ and a patience of 50 epochs; and (ii) adaptive learning-rate scheduling, reducing the learning rate by a factor of 0.5 when the validation loss plateaued for 25 epochs. The batch size was set to 8, and the initial learning rate was 3 × 10^−4^.

### 2.7. Model Evaluation

To assess classification performance on the prediction dataset, four metrics were computed: balanced accuracy (%), precision (%), recall (%), and F1-score. Their mathematical definitions are given in Equations (5)–(8).

Accuracy = [(TP/TP + FN) + (TN/TN + FP)] × 0.5 × 100,(5)

Precision = (TP/TP + FP) × 100,(6)

Recall = (TP/TP + FN) × 100,(7)

F1-score = [(2 × Precision × Recall)/(Precision + Recall)].(8)

In Equations (5)–(7), TP, TN, FP, and FN signify the true positive, true negative, false positive, and false negative. These scalars were generated through the confusion matrix [52]. In this study, accuracy denotes the proportion of correctly classified samples. Precision reflects the fraction of predicted positive samples that are correctly classified, while recall (sensitivity) indicates the proportion of actual positive samples that are correctly identified by the model. The F1-score is defined as the harmonic means of precision and recall, providing a balanced measure of classification performance [53]. The evaluation metrics were calculated utilizing the library provided by the Scikit-Learn (average = ’weighted’).

### 2.8. Software and Operating System

Data visualization was performed in Python (v3.12.10) using Microsoft Visual Studio Code (v1.108.1; Microsoft Corporation, Redmond, WA, USA) together with the ‘Matplotlib’ and ‘Seaborn’ libraries. All data analyses were carried out on a Windows 11 Pro (64-bit) operating system running on an Intel^®^ Core™ Ultra 7 265K processor (3.90 GHz) and equipped with an NVIDIA GeForce RTX 3080 Ti graphics processing unit.

## 3. Results and Discussion

### 3.1. Spectral Analysis

**Figure 7:**
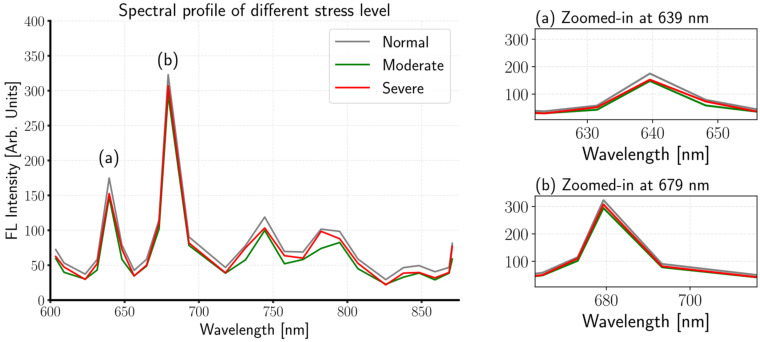
Representatives mean spectra of each class are shown, together with magnified fluorescence spectra at two specific wavelength regions (639 and 679 nm). In the figure, FL means fluorescence.

The fluorescence spectra extracted from the region of interest (ROI) spanning 603–870 nm are shown in Figure 7 and closely match the spectral response reported in [54]. In contrast to Vis-NIR spectroscopy [55], where this region is mainly dominated by absorption features associated with water and other constituents (e.g., soluble sugars), the fluorescence signal primarily reflects chlorophyll emission, as also supported by previous studies [27]. Consistent to the finding of [26], normal plants exhibited higher fluorescence intensity than stressed plants, which is indicative of chlorophyll-related changes under stress [56]. Notably, the most pronounced differences were observed around 639 and 679 nm, as highlighted in the magnified spectra.

### 3.2. Cross-Validation Results

Using the calibration dataset to learn the relationship between the fluorescence spectra and their corresponding class labels, all four models achieved strong performance, with accuracies ranging from 85.9% to 89.2% (Table 2). Among them, G-SVM delivered the highest accuracy (89.2%), outperforming LDA, QDA, and the deep-learning model. Notably, the deep learning yielded the lowest accuracy (85.9%), which was contrary to our initial expectation.

Moreover, the performance of cross-validation yielded all metrics ranging from 82.2 ± 2.0% to 86.2 ± 1.9%. Among them, LDA achieved the highest mean cross-validation accuracy of 85.1%, precision of 85.3 ± 3.0%, recall of 85.1 ± 2.9%, and F1-score of 85.0 ± 3.0, comparable to the existing literature [25,57,58].

**Figure 8:**
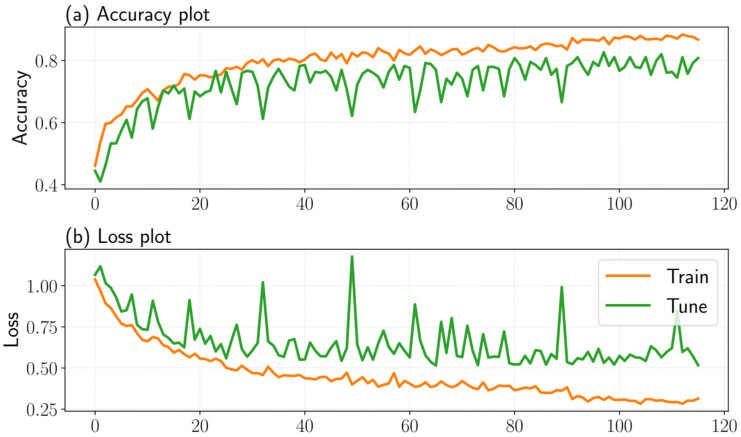
Accuracy (**a**) and loss (**b**) plots during the deep-learning training stage.

Furthermore, although the earlier discussion indicated that the deep learning was the weakest classifier on the single split evaluation, its performance improved under 10-fold cross-validation. In fact, the statistical analysis showed that the deep learning achieved a significantly higher accuracy across folds, yielding the highest mean cross-validation accuracy, precision, recall, and F1-score of 86.2 ± 1.9%. Although a larger number of epochs were initially planned for the training stage, the application of early stopping terminated the training after 116 epochs, corresponding to the minimum validation loss. Figure 8 illustrates the training and validation accuracy and loss curves over 116 epochs. A slight overfitting behavior is observed, as indicated by the divergence between the training and validation trends at later epochs.

### 3.3. Evaluation of the Model Using Prediction Dataset

**Figure 9:**
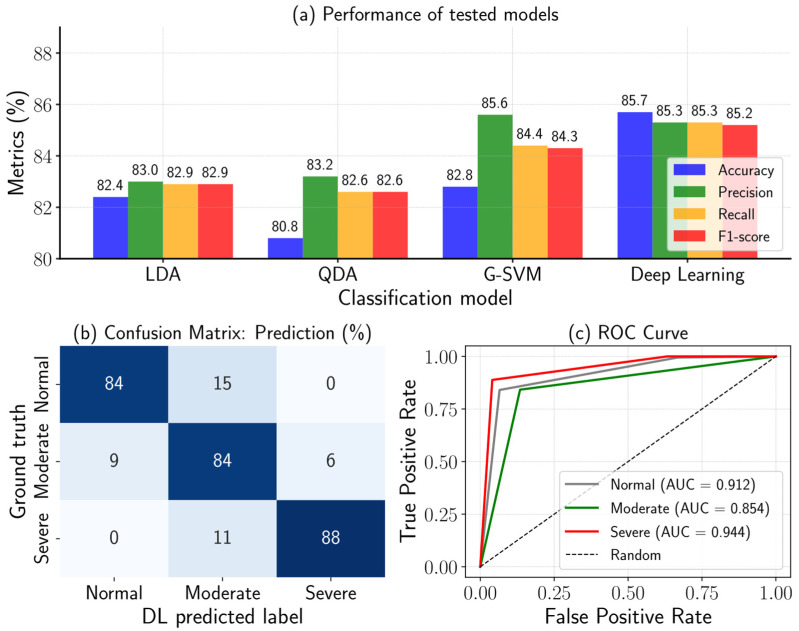
Summary of classification metrics of four models using prediction dataset (**a**), normalized prediction confusion matrix of deep learning (**b**), and the ROC curve (**c**).

Figure 9a presents a summary of the classifiers’ performance on the prediction dataset. Compared with the results reported in Table 2, several classifiers, namely LDA, QDA, and G-SVM, exhibited signs of overfitting, as their performance decreased when evaluated on the new data. This finding suggests that the classical machine-learning models used in this study had limited generalization capability to the prediction dataset.

In the confusion matrix (Figure 9b), the proportions of correctly and incorrectly classified samples are shown. Compared with the other models developed in this study (Table 3), all approaches exhibited a similar limitation, namely misclassification between adjacent classes (normal → moderate and/or moderate → severe). Additionally, the receiver operating characteristic (ROC) curves (Figure 9c) indicate that the model achieved moderate-to-strong discriminative performance, with AUC values ranging from 0.854 to 0.944. Among the three stress levels, the severe stress class yielded the highest AUC, suggesting that the model most reliably identifies severe cold-stress symptoms. In contrast, the moderate stress class showed the lowest AUC, implying greater spectral overlap with adjacent classes. This observation is consistent with the confusion matrix in Figure 9b, where most misclassifications occur between the moderate group and the other stress levels.

This behavior likely indicates partial overlap among these classes, which reduces the separability of their spectral signatures and makes accurate discrimination more challenging. In addition, variability in the spatial distribution of spectral responses may also have contributed to these errors. Because a single illumination source was used, non-uniform pixel intensity across the leaf surface may have occurred. Furthermore, spectral features are closely linked to non-uniform chloroplast distribution [59,60,61]. Consequently, the associated changes may not be homogeneously distributed across the leaf, leading to within-class variability and increased confusion between neighboring severity levels. Nonetheless, the deep learning consistently yielded a lower misclassification error compared with the other models [62,63,64].

### 3.4. Gradient-Weighted Class Activation Mapping (Grad-CAM)

**Figure 10:**
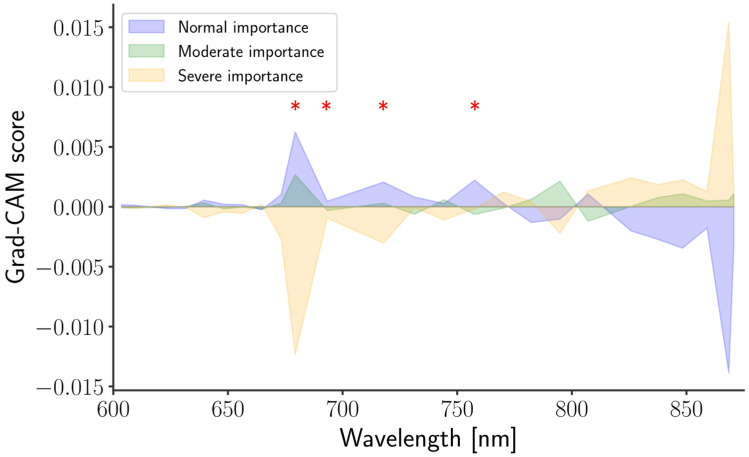
Area plot showing the Grad-CAM scores of each class across the wavelengths. The red asterisks identify the wavelengths with higher contributions (*p* < 0.05): 679, 693, 717, and 757 nm.

Grad-CAM [65] is a widely used technique for improving the interpretability of deep-learning models. Although it was originally developed for image-based data, it has also been shown to be effective for one-dimensional inputs, such as the spectra [66,67,68]. The Grad-CAM visualizations obtained in this study are presented in Figure 10 that was calculated using the correctly predicted samples. By further examining the ANOVA results, four wavelengths (679, 693, 717, and 757 nm; denoted by red asterisks) were identified as exhibiting the most significant class-dependent differences in Grad-CAM scores. At these wavelengths, samples in the severe group showed predominantly negative Grad-CAM values, whereas the normal and moderate groups were characterized by positive values, which is contrary to that of ref. [53].

These wavelengths fall within the chlorophyll-related emission region, suggesting that severe stress substantially alters the spectral response. This interpretation is further supported by previous studies reporting that freezing temperatures can destabilize PSII activity in potato [69], reduce chlorophyll biosynthesis [70] and inhibit photosynthetic carbon metabolism [71] in rice seedlings, as well as related findings in ref. [72]. Nevertheless, this interpretation should be accompanied by complementary wet-chemistry measurements.

### 3.5. Generation of Classification Maps

**Figure 11:**
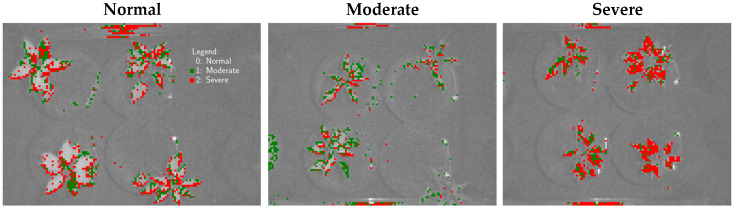
Classification maps produced by a deep learning using multispectral hypercube data for different stress levels.

Leveraging the benefits of using multispectral imaging system over conventional spectroscopy techniques, the pixel-level prediction can be made available [73]. Because the deep-learning output was one hot-encoded matrix, it must be converted to integer class labels to enable the construction of classification maps. The resulting classification maps for the different treatments are shown in Figure 11. Based on model evaluation, deep learning was selected as the best-performing model. However, because its overall predictive performance was below 86%, the expected misclassification rate is approximately 14%. This uncertainty is reflected in the classification maps; for example, although pixels in the normal group are expected to be labeled as class 0, a subset of pixels is incorrectly assigned to classes 1 and 2 (moderate and severe). Similar mislabeling is also observed in the moderate and severe groups. These events were likely caused by spectral overlaps among classes. Moreover, we suspect that the deep-learning model (as well as the other models) was developed using average leaf spectra, which may have reduced within-class variability and contributed to the observed confusion [74].

## 4. Conclusions

The feasibility of snapshot multispectral fluorescence imaging for nondestructive cold-stress detection in pepper plants was investigated in this study. Because spectral preprocessing did not improve performance, only the raw fluorescence spectra were used for model development. Three classical machine learning models (LDA, QDA, and G-SVM) and a deep-learning model were developed to classify three stress levels. Cross-validation indicated the deep learning achieved the highest and statistically distinct accuracy compared with the other models (86.2 ± 1.9%). The models were further evaluated using an independent prediction dataset, where the deep learning again provided the best performance for three-class classification, with accuracy of 85.7%, precision of 85.3%, recall of 85.3%, F1-score of 85.2. These outcomes were consistent with the generated classification maps, which still showed moderate misclassification in certain regions.

Although the present study suggests that deep learning outperforms classical machine-learning methods, several limitations should still be acknowledged. These include the need to further examine how modifications to the network layers influence prediction accuracy, particularly whether greater architectural complexity leads to improved or diminished performance. Moreover, additional hyperparameter-optimization trials may further enhance predictive accuracy. Other strategies, such as data fusion, may also offer improved model performance.

## Figures and Tables

**Table 1:** Detailed of the deep-learning structure and number of parameters on each layer involved in this work.

Block	Layer	Output Shape
Input layer	Input spectra	None, 25, 1
1D-CNN	Conv1D #1	None, 25, 128
	Conv1D #2 + BN + ReLU	None, 25, 128
RNN	Encoder—GRU	(None, 64), (None, 64)
	Context vector	(None, 25, 64)
	Decoder—GRU	(None, 64)
MLP #1	Dense #1 + ReLU	None, 64
MLP #2	Dense #2	None, 64
Output layer	Softmax	None, 3

**Table 2:** Comparison of models’ performance during calibration and cross-validation (10-fold).

Model	Calibration				Cross-Validation			
	Accuracy (%)	Precision (%)	Recall (%)	F1-Score	Accuracy (%)	Precision (%)	Recall (%)	F1-Score
LDA	86.6	86.4	86.4	86.4	85.1 ± 2.7	85.3 ± 3.0	85.1 ± 2.9	85.0 ± 3.0
QDA	88.4	88.6	88.4	88.6	82.6 ± 3.5	83.3 ± 3.4	82.6 ± 3.6	82.7 ± 3.6
G-SVM	89.2	88.0	88.2	88.2	82.2 ± 2.0	84.0 ± 2.0	82.2 ± 2.1	82.4 ± 2.1
DL ^**)^	85.9	85.4	85.4	85.2	86.2 ± 1.9	86.2 ± 1.9	86.2 ± 1.9	86.1 ± 1.9

^**)^ DL = deep learning.

**Table 3:** Normalized confusion matrices for LDA, QDA, and G-SVM.

Model	Ground Truth\\ Prediction	Calibration			Prediction		
		0	1	2	0	1	2
LDA	0	91.2	8.8	0	79.0	20.0	0
	1	12.1	80.2	7.7	8.0	84.0	6.0
	2	0.2	11.5	88.3	0	16.0	83.0
QDA	0	90.8	9.2	0	78.0	21.0	0
	1	8.4	86.2	5.4	7.0	88.0	3.0
	2	0.4	11.2	88.3	0	23.0	75.0
G-SVM	0	88.1	11.9	0.0	75.5	24.5	0.0
	1	6.8	92.3	0.9	6.7	92.7	0.6
	2	0	18	82	0	22	78

## Data Availability

Data will be made available on request.
